# Supplementary figures and images for: Cezanne (OTUD7B) regulates HIF-1α homeostasis in a proteasome-independent manner
Source: EMBO Rep. 2014 Oct 29;15(12):1268–77. doi: 10.15252/embr.201438850 (PMC4264929; doi:10.15252/embr.201438850)

**Figure S1**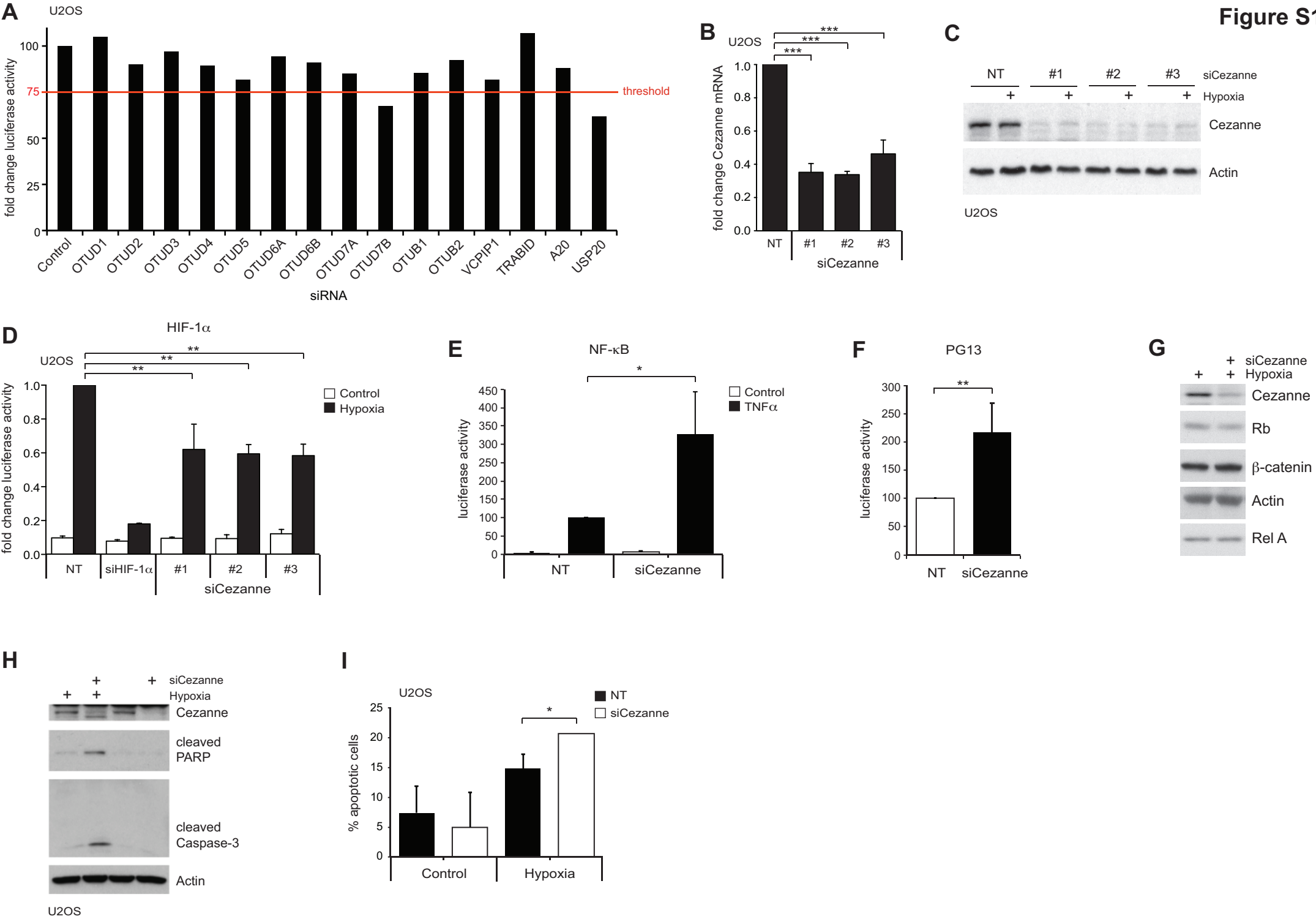

Supplement: Supplementary file 1 — Supplementary Figure S1 [file embr0015-1268-sd1.pdf]

Figure S2

A

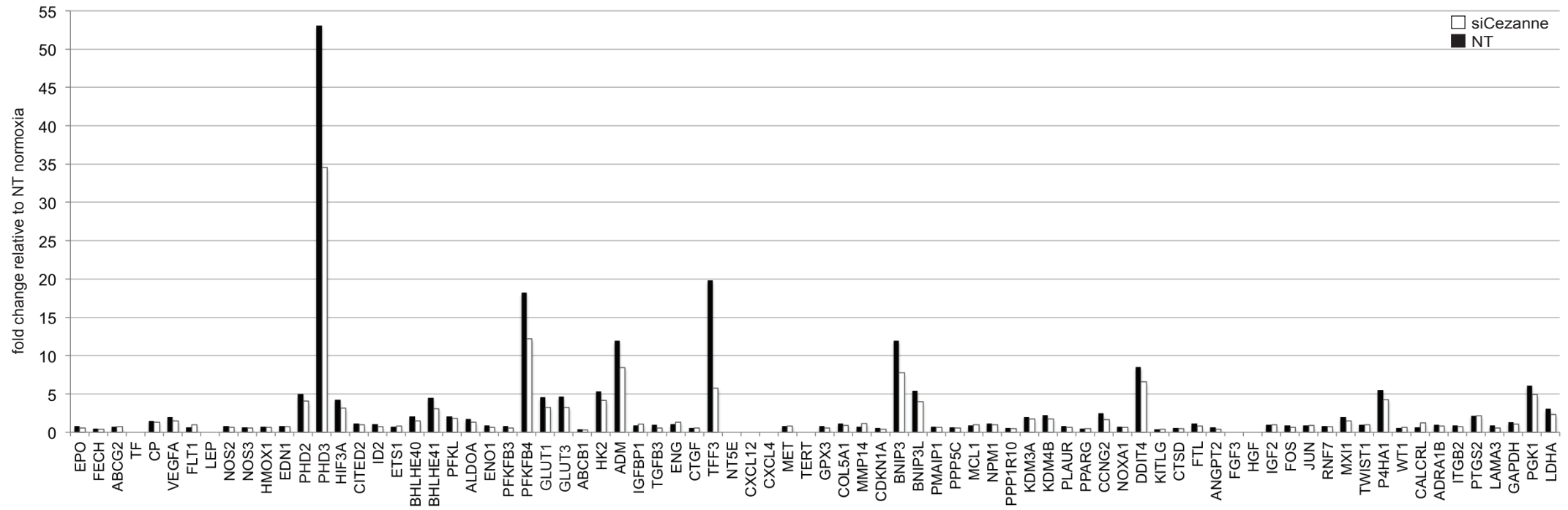

B

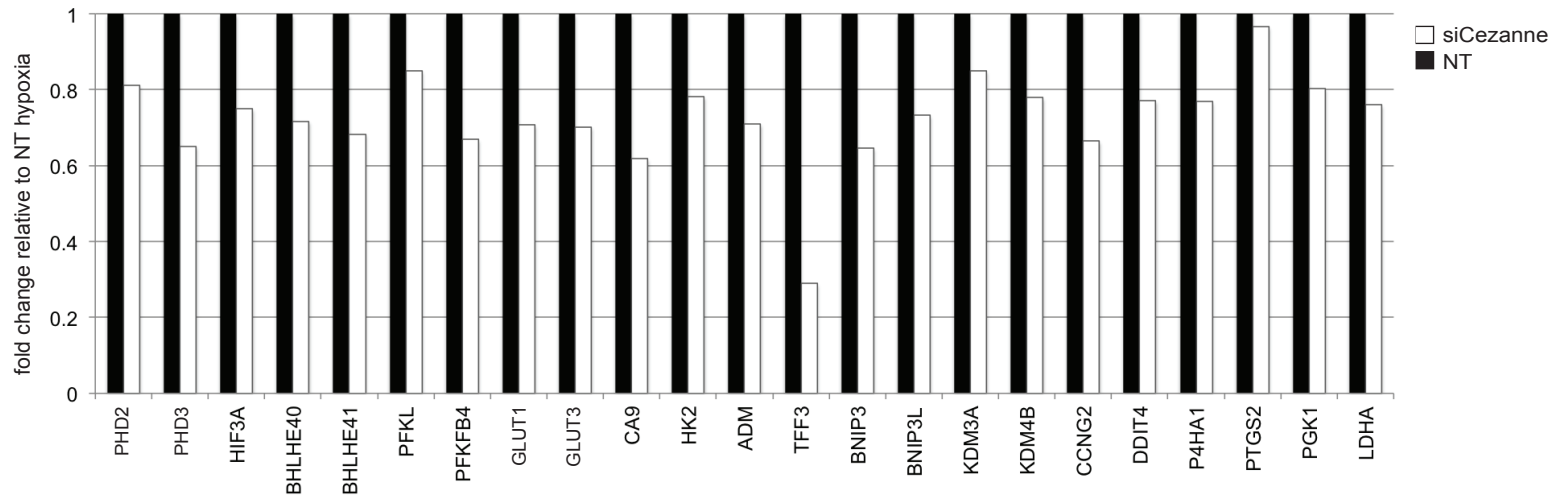

Supplement: Supplementary file 2 — Supplementary Figure S2 [file embr0015-1268-sd2.pdf]

Figure S3

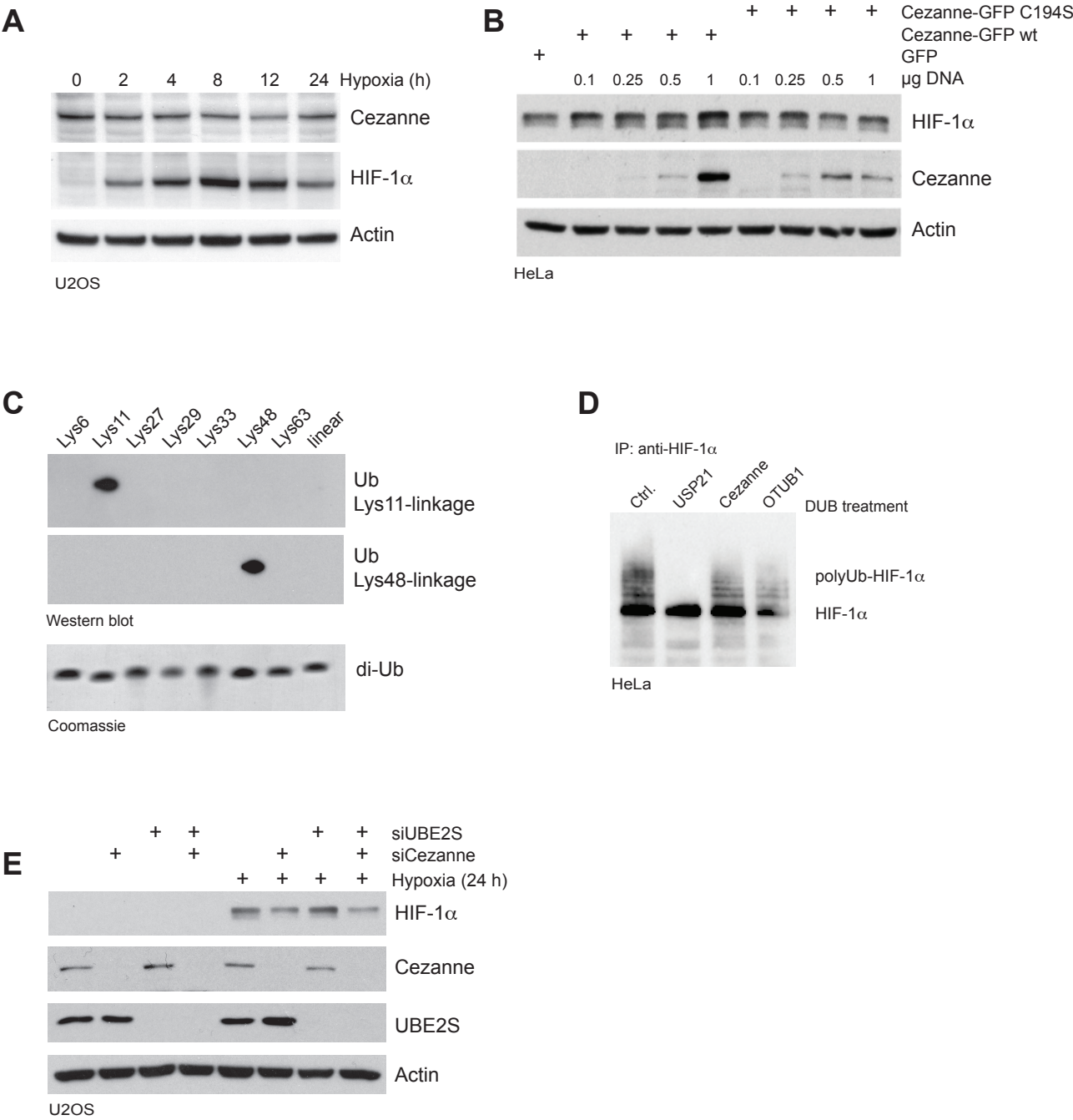

Supplement: Supplementary file 3 — Supplementary Figure S3 [file embr0015-1268-sd3.pdf]

**Figure S4**

**A**

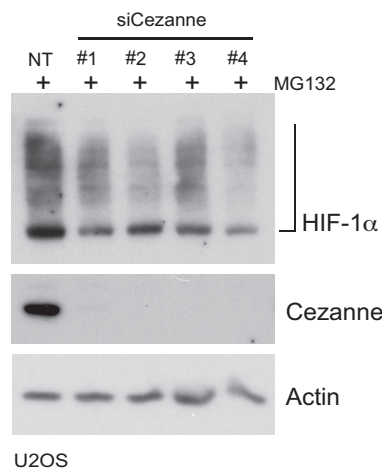

**B**

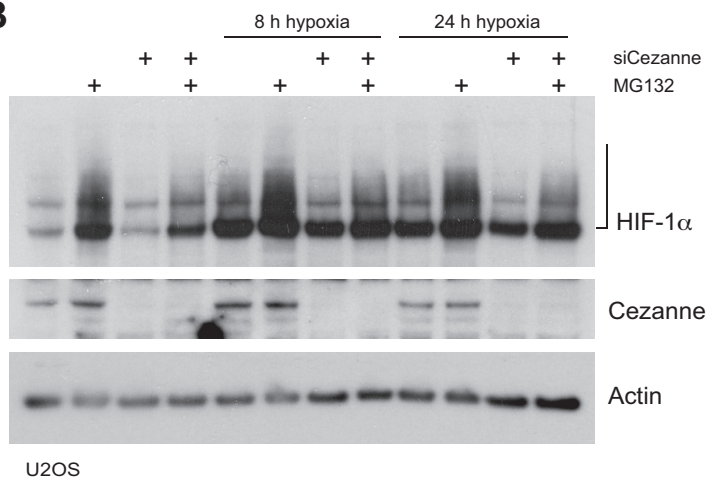

**C**

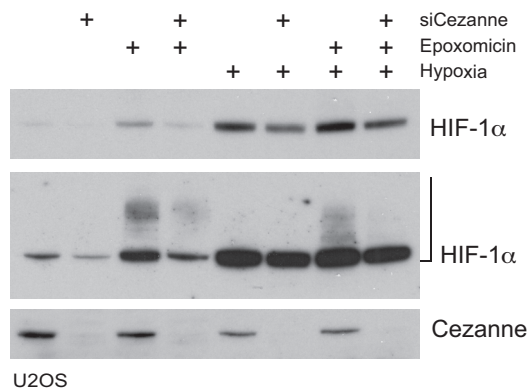

**D**

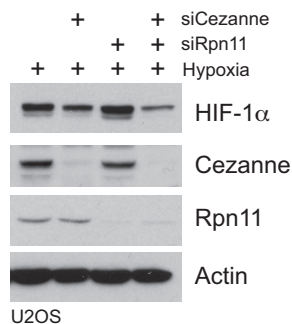

**E**

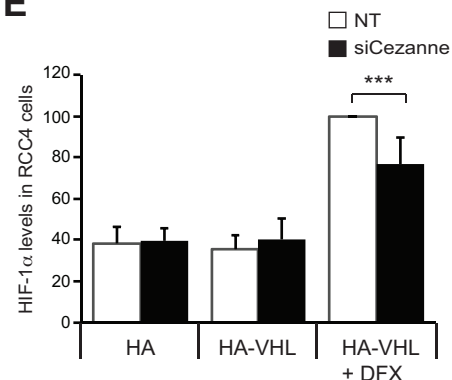

**G**

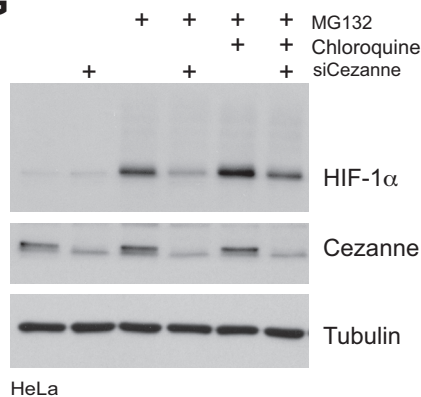

**H**

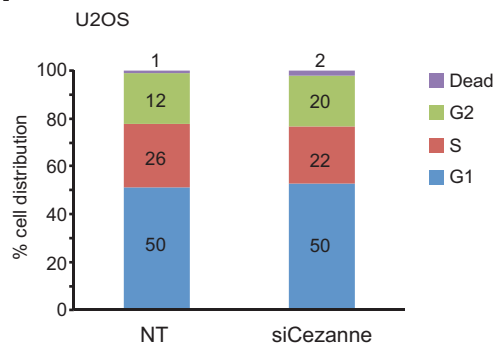

Supplement: Supplementary file 4 — Supplementary Figure S4 [file embr0015-1268-sd4.pdf]
