# Supplementary material for: Cezanne (OTUD7B) regulates HIF-1α homeostasis in a proteasome-independent manner
Source: EMBO Rep. 2014 Oct 29;15(12):1268–77. doi: 10.15252/embr.201438850 (PMC4264929; doi:10.15252/embr.201438850)
Supplement: Supplementary file 5 — Supplementary Information [file embr0015-1268-sd5.pdf]

Cezanne (OTUD7B) regulates HIF-1α homeostasis in a proteasome-independent manner

Supplementary Information

Anja Bremm[1,2,*], Sonia Moniz[3], Julia Mader[1], Sonia Rocha[3,*] and David Komander[2,*]

[1] Buchmann Institute for Molecular Life Sciences, Institute of Biochemistry II, Goethe University, Max-von-Laue-Str. 15, 60438 Frankfurt (Main), Germany

[2] Medical Research Council Laboratory of Molecular Biology, Francis Crick Avenue, Cambridge Biomedical Campus, Cambridge, CB2 0QH, UK

[3] College of Life Sciences, Centre for Gene Regulation and Expression, University of Dundee, Dundee, DD1 5EH, UK

* Correspondence to: Anja Bremm (bremm@em.uni-frankfurt.de), Sonia Rocha (s.rocha@dundee.ac.uk), David Komander (dk@mrc-lmb.cam.ac.uk)

Figure legends

Figure S1. Cezanne regulates HIF-1α - siRNA deconvolution and controls.

A siRNA knockdown of 14 OTU-family DUBs in U2OS cells treated with hypoxia (1% oxygen) for 24 h. Depletion of Cezanne and USP20 decreased HRE-luciferase activity to similar extends compared to control cells. The presented screen was conducted once in triplicate (Z-score >0.8). **B, C** Transfection of three different Cezanne siRNA oligonucleotides decreased Cezanne mRNA (B) and protein levels (C). **D** Reporter gene assay showing that depletion of Cezanne resulted in decreased HIF-1α-dependent luciferase activity. **E, F** In contrast, loss of Cezanne increased activity of the transcription factors NF-κB and p53 (PG13 reporter). **G** Protein levels of Rb, β-catenin, and the NF-κB subunit RelA are not affected by loss of Cezanne. **H, I** siRNA knockdown of Cezanne resulted in upregulated cleaved-PARP and cleaved-Caspase 3 levels (H), and an increase in the number of apoptotic cells in hypoxia as seen by Annexin V staining (I). (If not specified differently, all experiments were performed three times; bar graphs represent the mean plus standard deviation of these independent experiments. P values were calculated using Student's t test (* < 0.1, **p < 0.01, ***p < 0.001).)

Figure S2. Global effects on HIF-1α target gene expression.

A Cezanne-depleted and control U2OS cells were treated with hypoxia (24h) and expression of 81 HIF-target genes was analysed by RT-PCR using a customised PCR screen. Threshold cycle (C_T) for each well was calculated using the Rotor-Gene software (QIAGEN) after threshold was manually defined.

Subsequently, fold change was calculated by the web-based PCR Array data analysis tool provided by Qiagen. Data are shown as fold change when compared to untreated, NT control. **B** 23 genes out of the 81 genes analysed in (A) were induced more than 2-fold in hypoxia treated cells. Hypoxia-dependent induction of these genes was reduced in Cezanne-depleted cells. mRNA levels are shown relative to hypoxia-treated control (NT) cells. See **Table S2**. (Bar graphs represent the mean of two independent experiments.)

Figure S3. Controls.

A U2OS cells were treated with hypoxia and Cezanne protein levels were monitored at various time points. **B** Overexpression of GFP-tagged wild-type or inactive Cezanne in hypoxia-treated U2OS cells affected HIF-1α levels in a dose-dependent manner. **C** Identical amounts of diubiquitin of all eight linkage types (Coomassie stained, bottom panel) were blotted and incubated with Lys11-linkage specific antibody (top panel) or Lys48-linkage specific antibody (middle panel). **D** Ubiquitin chain restriction (UbiCRest) analysis [1] of polyubiquitinated HIF-1α using non-specific USP21 as a positive control, as well as the Lys11-specific Cezanne catalytic domain, and the Lys48-specific OTUB1, both of which reduce HIF-1α poly-ubiquitination. **D** Loss of Cezanne also resulted in lower HIF-1α protein levels when the Lys11 linkage-specific E2 enyzme UBE2S was co-depleted. (All experiments were performed three times.)

Figure S4. Effect of Cezanne on HIF-1α degradation

A Reduced HIF-1α protein levels upon Cezanne knockdown in MG132-treated cells was observed with four different siRNA oligonucleotides. **B** Cezanne-depleted and control U2OS cells were treated with hypoxia for 8 h or 24 h and directly lysed in SDS sample buffer. Knockdown of Cezanne decreased HIF-1α protein levels in normoxia and hypoxia. **C** Inhibition of proteasome activity by epoxomicin did not rescued HIF-1α levels in Cezanne-depleted cells neither under normoxia nor under hypoxia. **D** Co-depletion of Cezanne and the proteasome subunit Rpn11 resulted in decreased HIF-1α levels. **E** VHL-negative RCC4 cells expressed equal HIF-1α levels in the presence and absence of Cezanne. Reconstitution of VHL rendered RCC4 cells sensitive again for HIF-1α regulation by Cezanne, i.e. in HA-VHL expressing RCC4 cells HIF-1α levels were decrease by approx. 25% when Cezanne was depleted. Quantification of four independent experiments using ImageJ software. **F** Combined inhibition of proteasome and lysosome activity by MG132 and chloroquine did not fully rescue HIF-1α levels in Cezanne-depleted cells (lane 5-6). **G** Knockdown of Cezanne did not alter cell cycle distribution. (If not specified differently, all experiments were performed three times; bar graphs represent the mean plus standard deviation of these independent experiments. P values were calculated using Student's t test (***$p < 0.001$).)

Table S1. HIF-target genes analysed in PCR screen

Uniprot	Gene name	Protein name
P08183	ABCB1	Multidrug resistance protein 1
Q9UNQ0	ABCG2	ATP-binding cassette sub-family G member 2
P35318	ADM	ADM
P35368	ADRA1B	Alpha-1B adrenergic receptor
P04075	ALDOA	Fructose-bisphosphate aldolase A
O15123	ANGPT2	Angiopoietin-2
O14503	BHLHB2	Basic helix-loop-helix family, member e40
Q9C0J9	BHLHB3	Basic helix-loop-helix family, member e41
Q12983	BNIP3	BCL2/adenovirus E1B 19 kDa protein-interacting protein 3
O60238	BNIP3L	BCL2/adenovirus E1B 19 kDa protein-interacting protein 3-like
Q16790	CAIX	Carbonic anhydrase IX
Q16602	CALCRL	Calcitonin gene-related peptide type 1 receptor
Q16589	CCNG2	Cyclin G2
P38936	CDKN1A	Cyclin-dependent kinase inhibitor 1
Q99967	CITED2	Cbp/p300-interacting transactivator 2
P20908	COL5A1	Collagen alpha-1(V) chain
P00450	CP	Ceruloplasmin
P29279	CTGF	Connective tissue growth factor
P07339	CTSD	Cathepsin D
P48061	CXCL12	Stromal cell-derived factor 1
P61073	CXCR4	C-X-C chemokine receptor type 4
Q9NX09	DDIT4/RTP801	DNA damage-inducible transcript 4 protein
Q9GZT9	EGLN1	Egl nine homolog 1 (PHD2)
Q9H6Z9	EGLN3	Egl nine homolog 3 (PHD3)
P05305	END1	Endothelin-1
P17813	ENG	Endoglin
P06733	ENO1	Alpha-enolase
P01588	EPO	Erythropoietin
P14921	ETS1	Protein C-ets-1
P22830	FECH	Ferrochelatase, mitochondrial
P11487	FGF3	Fibroblast growth factor 3
P17948	FLT1	Vascular endothelial growth factor receptor 1
P01100	FOS	Proto-oncogene c-Fos
P02792	FTL	Ferritin light chain
P04406	GAPDH	Glyceraldehyde-3-phosphate dehydrogenase
P22352	GPX3	Glutathione peroxidase 3
P14210	HGF	Hepathocyte growth factor
Q9Y2N7	HIF3A	Hypoxia-inducible factor 3-alpha
P52789	HK2	Hexokinase-2
P09691	HMOX1	Heme oxygenase 1
Q02363	ID2	DNA-binding protein inhibitor ID-2
P01344	IGF2	Insulin-like growth factor II
P08833	IGFBP1	Insulin-like growth factor-binding protein 1
P05107	ITGB2	Integrin beta-2
P05412	JUN	Transcription factor AP-1
Q9Y4C1	KDM3A	Lysine-specific demethylase 3A
O94953	KDM4B	Lysine-specific demethylase 4B
P21583	KITLG	Kit ligand / Stem cell factor

Q16787	LAMA3	Laminin subunit alpha-3
P00338	LDHA	L-lactate dehydrogenase A chain
P41159	LEP	Leptin
Q07820	MCL1	Induced myeloid leukemia cell differentiation protein Mcl-1
P08581	Met	Hepatocyte growth factor receptor
P50281	MMP14	Matrix metalloproteinase-14
P50539	MXI1	Max-interacting protein 1
P35228	NOS2A	Nitric oxide synthase, inducible
P29474	NOS3	Nitric oxide synthase, endothelial
Q86UR1	NOXA1	NADPH oxidase activator 1
P06748	NPM1	Nucleophosmin
P21589	NT5E	Ecto-5'-nucleotidase
P13674	P4HA1	Prolyl 4-hydroxylase subunit alpha-1
P17858	PFK	6-phosphofructokinase, liver type
Q16875	PFKFB3	6-phosphofructo-2-kinase/fructose-2,6-biphosphatase 3
Q16877	PFKFB4	6-phosphofructo-2-kinase/fructose-2,6-biphosphatase 4
P00558	PGK1	Phosphoglycerate kinase 1
Q03405	PLAUR	Urokinase plasminogen activator surface receptor
Q13794	PMAIP1	Phorbol-12-myristate-13-acetate-induced protein 1
P37231	PPARG	Peroxisome proliferator-activated receptor gamma
Q96QC0	PPP1R10	Serine/threonine-protein phosphatase 1 regulatory subunit 10
P53041	PPP5C	Serine/threonine-protein phosphatase 5
P35354	PTGS2	Prostaglandin G/H synthase 2
Q9UBF6	RNF7	RING-box protein 2 (RBX2)
P11166	SLC2A1	Solute carrier family 2, facilitated glucose transporter member 3
P11169	SLC2A3	Solute carrier family 2, facilitated glucose transporter member 3
O14746	TERT	Telomerase reverse transcriptase
P02787	TF	Serotransferrin
Q07654	TFF3	Trefoil factor 3
P02786	TFRC	Transferrin receptor protein 1
P10600	TGFB3	Transforming growth factor beta-3
Q15672	TWIST1	Twist-related protein 1
P15692	VEGFA	Vascular endothelial growth factor A
P19544	WT1	Wilms tumor protein

Table S2. Altered HIF-target gene expression in Cezanne-depleted U2OS cells

Listed genes (column 1) were induced more than 2-fold compared to non-targeting control. The absolute values for induction (column 2) compared to the absolute values in Cezanne-depleted cells (column 3) are shown. Column 4 shows the % reduction in target gene expression induced by Cezanne knockdown. Data is represented in **Figure S2**.

Gene	NT	siCezanne	siCezanne % reduction
ADM	11.92	8.46	29.04
BHLHE40	2.03	1.45	28.30
BHLHE41	4.47	3.05	31.70
BNIP3	11.96	7.73	35.38
BNIP3L	5.45	3.99	26.80
CA9	663.98	410.15	38.23
CCNG2	2.46	1.64	33.57
DDIT4	8.52	6.57	22.89
EGLN1	4.96	4.03	18.77
EGLN3	53.08	34.54	34.93
HIF3A	4.20	3.15	25.00
HK2	5.31	4.16	21.81
KDM3A	2.01	1.71	15.03
KDM4B	2.27	1.77	22.08
LDHA	3.06	2.33	23.95
PFKFB4	18.19	12.17	33.10
PFKL	2.08	1.77	15.03
PGK1	6.06	4.87	19.62
PTGS2	2.18	2.11	3.41
P4HA1	5.46	4.20	23.16
SLC2A1	4.56	3.23	29.29
SLC2A3	4.66	3.27	29.78
TFF3	19.84	5.74	71.08

Materials and Methods

siRNA oligonucleotides

siRNA oligonucleotides were synthesized by Thermo Scientific (Non-targeting siRNA control, D-001810-01; SMARTpool ZA20D1 (OTUD7B/ Cezanne), L-008670-00; Set of 4 siRNA ZA20D1 (OTUD7B/ Cezanne), LQ-008670-00; SMARTpool UBE2S, L-009707-00); Eurofins MWG Operon (HIF-1, CUG AUG ACC AGC AAC UUG A [1]); (Rpn11, UUA CAA UAA GGC UGU AGA A); or Life Technologies (VCP/p97, a kind gift from L. James, MRC Laboratory of Molecular Biology, Cambridge, UK).

Antibodies used for immunoblots

HIF-1α (MAB1536, R&D Systems), Cezanne (custom antibody, Eurogentec), beta Actin (ab8227, Abcam), LC3B (2775, Cell Signaling), cleaved PARP (Asp214) (9541, Cell Signaling), cleaved Caspase-3 (9664, Cell Signaling), PHD3 (A300-327A, Bethyl Labs), BNIP3 (ab10433, Abcam), HIF-2α (PA1-16510, Thermo Pierce), Tubulin (2125, Cell Signaling), CUL-2 (sc-166506, Santa Cruz), Factor Inhibiting HIF-1 (NB100-428, Novus Biologicals), Von Hippel Lindau (NB100-485SS, Novus Biologicals), beta-catenin (4270, Cell Signalin), Rb (9309, Cell Signaling), RelA (sc-372, Santa Cruz), Ubiquitin (07-375, Millipore), Ubiquitin K11 linkage, clone 2A3/2E6 (MABS107, Millipore), Ubiquitin, Lys48-specific, clone Apu2 (05-1307, Millipore), UBE2S, (11878, Cell Signaling), p97 ATPase (MA1-21412, Thermo Scientific), GFP (sc-8334Santa Cruz), Rpn11 (Cell Signaling), Normal Rabbit IgG (2729, Cell Signaling)

Immunoprecipitation of GFP-tagged Cezanne

HEK293 cells were transfected with 4 µg pOPIN-GFP-Cezanne plasmid DNA per 10 cm culture dish using GeneJuice (Merck Biosciences) according to manufacturer's instructions. Cells were lysed in 10 mM Tris-HCl (ph 7.5), 150 mM NaCl, 0.5% (v/v) IGEPAL® CA-630, 100 mM NaF and 1 tablet/10 ml *Complete*, Mini, EDTA-free protease inhibitors (Roche). Cleared cell lysate was rotated at 4°C for 2 h with GFP-Trap®-A agarose beads. GFP-Trap®-Cezanne complex was washed twice with PBS, once with 1× DUB buffer (50 mM NaCl, 50 mM Tris (pH 7.5) and 5 mM DTT) and immediately used for deubiquitination assays.

Deubiquitination assay

Full-length GFP-tagged Cezanne was isolated from two 10 cm culture dishes HEK293 cells (see above) and divided into eight different reaction tubes. Cezanne-GFP-Trap®-agarose complex was spun down, resuspended in 30 µl 1× DUB buffer (50 mM NaCl, 50 mM Tris (pH 7.5) and 5 mM DTT) containing 2 µg diubiquitin and incubation at 37°C. 10 µl were taken immediately (time point zero) and after 60 min, and reactions were stopped by adding 10 µl LDS sample buffer (Life Technologies). Ubiquitin cleavage was detected by silver staining using the Silver Stain Plus kit (BioRad).

Ubiquitin chain restriction analysis (UbiCRest)

Ubiquitinated HIF-1α was immunoprecipitated from HeLa cells treated with 20 µM MG132 for 4 h as described above. Immobilized antigen-antibody complex was washed with 1× DUB buffer (see above), distributed into four different

tubes, spun down and resuspended again in 25 µl 1× DUB buffer. 5 µl of diluted DUBs were mixed with the substrate and incubated for 60 min at 37 °C before the reaction was stopped by adding 10 µl 4× LDS sample buffer. Samples were resolved on 4%-12% SDS-PAGE gradient gels and analysed by western blotting.

References

1. van Uden P, Kenneth NS, Rocha S (2008) Regulation of hypoxia-inducible factor-1α by NF-κB. *Biochem J* **412**: 477–484.
